# Sex-related and tissue-specific effects of tobacco smoking on brain atrophy: assessment in a large longitudinal cohort of healthy elderly

**DOI:** 10.3389/fnagi.2014.00299

**Published:** 2014-11-03

**Authors:** Quentin Duriez, Fabrice Crivello, Bernard Mazoyer

**Affiliations:** ^1^Life Sciences, University of Bordeaux, Neurofunctional Imaging Group (GIN) UMR5296Bordeaux, France; ^2^Centre National de la Recherche Scientifique, Neurofunctional Imaging Group (GIN) UMR5296Bordeaux, France; ^3^Commisariat à l'Energie Atomique, Neurofunctional Imaging Group (GIN) UMR5296Bordeaux, France

**Keywords:** tobacco, aging, sex, brain atrophy, gray matter, white matter, hippocampus, MRI

## Abstract

We investigated the cross-sectional and longitudinal effects of tobacco smoking on brain atrophy in a large cohort of healthy elderly participants (65–80 years). MRI was used for measuring whole brain (WB), gray matter (GM), white matter (WM), and hippocampus (HIP) volumes at study entry time (baseline, *N* = 1451), and the annualized rates of variation of these volumes using a 4-year follow-up MRI in a subpart of the cohort (*N* = 1111). Effects of smoking status (never, former, or current smoker) at study entry and of lifetime tobacco consumption on these brain phenotypes were studied using sex-stratified AN(C)OVAs, including other health parameters as covariates. At baseline, male current smokers had lower GM, while female current smokers had lower WM. In addition, female former smokers exhibited reduced baseline HIP, the reduction being correlated with lifetime tobacco consumption. Longitudinal analyses demonstrated that current smokers, whether men or women, had larger annualized rates of HIP atrophy, as compared to either non or former smokers, independent of their lifetime consumption of tobacco. There was no effect of smoking on the annualized rate of WM loss. In all cases, measured sizes of these tobacco-smoking effects were of the same order of magnitude than those of age, and larger than effect sizes of any other covariate. These results demonstrate that tobacco smoking is a major factor of brain aging, with sex- and tissue specific effects, notably on the HIP annualized rate of atrophy after the age of 65.

## Introduction

With the aging of the general population and the associated economical and social burden of age-related disorders, it has become critical to characterize normal brain aging and to identify and quantify factors that may accelerate brain aging and/or lead to age-related neurological disorders. Brain tissue volumes, whether global or regional (notably hippocampus), are endophenotypes easily derivable from magnetic resonance imaging (MRI) that have been shown to be sensitive to aging and good predictors of dementia and cognitive decline in elderly individuals (Miller et al., 1980; Braak and Braak, 1998; Good et al., 2001; Den Heijer et al., 2002; Fjell and Walhovd, 2010). Using such brain phenotypes, many studies have investigated factors of morphological brain aging such as sex (Coffey et al., 1998; Resnick et al., 2003; Lemaître et al., 2005b; Abe et al., 2010; Hua et al., 2010; O'Dwyer et al., 2012; Ryan et al., 2014), genetics (Lemaître et al., 2005a; Crivello et al., 2010; Boada et al., 2012; Stein et al., 2012; Ryan et al., 2014) or lifestyle (Coffey et al., 1999; Sabia et al., 2014; Shpanskaya et al., 2014; Umene-Nakano et al., 2014). Others have demonstrated the effect on brain atrophy of cardiovascular risk factors such as obesity (Driscoll et al., 2012; Xu et al., 2013; Debette et al., 2014, 2011; Franke et al., 2014), hypertension (Debette et al., 2011; Maillard et al., 2012; Peters, 2012; Beauchet et al., 2013; Franke et al., 2014), hypercholesterolemia (Tendolkar et al., 2012; Van Velsen et al., 2013; Franke et al., 2014), diabetes (Biessels et al., 2005; Debette et al., 2011; Cherbuin et al., 2012; Franke et al., 2014) and tobacco smoking (Enzinger et al., 2005; Ikram et al., 2008; Debette et al., 2011; Durazzo et al., 2012; Hoogendam et al., 2012).

Regarding the last factor, its effects on brain atrophy has recently received increased attention (Almeida et al., 2011; Durazzo et al., 2012, 2013; Pan et al., 2013; Fritz et al., 2014) because the consensus has been growing over the past years on the fact that it should be considered as a brain aging enhancer (Bernhard et al., 2007; Debette et al., 2011) and a risk factor for cognitive decline (Ott et al., 2004; Anstey et al., 2007; Starr et al., 2007; Sabia, 2012) and dementia (Ott et al., 1998; Anstey et al., 2007; Rusanen et al., 2011). Recent MRI studies have so looked for associations between tobacco consumption and either brain tissue global volumes (Enzinger et al., 2005; Ikram et al., 2008; Debette et al., 2011; Durazzo et al., 2012; Hoogendam et al., 2012), tissue local density (Brody et al., 2004; Gallinat et al., 2006; Almeida et al., 2008; Liao et al., 2010; Yu et al., 2011; Zhang et al., 2011; Morales et al., 2012), or white matter integrity (Paul et al., 2008; Debette et al., 2011; Umene-Nakano et al., 2014).

However, a solid consensus has not yet been reached for any of these morphological outcomes. Regarding global brain volumes, for instance, some authors have reported enhanced reduction (or accelerated rate of loss) of whole brain volume (WB, Ikram et al., 2008; Debette et al., 2011; Hoogendam et al., 2012), while others described no reduction or no accelerated rate of loss (Enzinger et al., 2005; Durazzo et al., 2012). As for gray matter (GM) regional densities, a meta-analysis of seven studies (Pan et al., 2013) found that a reduction in the anterior cingulate cortex of smokers was the only robust finding. Similar discrepancy in results were observed regarding white matter (WM) integrity, some showing reduced WM integrity in smokers compared to non-smokers (Debette et al., 2011; Umene-Nakano et al., 2014) while another study showed increased WM integrity in smokers (Paul et al., 2008).

Such discrepancies are likely to come from multiple sources, starting with small sample sizes, especially of current smokers, when dealing with cohorts of elderly, leading some studies to pool current and former smokers in their analyses while others did not. It must also be stressed that voxel-based studies are intrinsically subject to reduced statistical power given the very large number of brain phenotypes simultaneously analyzed. Another potential source of discrepancy is the choice of whole brain volume as a phenotype of interest. Indeed, gray and white matters have different dynamics during development, maturation and aging (Miller et al., 1980; Smith et al., 2007; Abe et al., 2010). Moreover, those two tissues have a highly different cellular and vascular composition, leaving the possibility of differential susceptibility to tobacco smoking, and calling for including gray and white volumes as additional phenotypes of interest. Finally, another factor that could lead to discrepancies is sex. There is evidence of sexual dimorphism in brain aging (Coffey et al., 1998; Good et al., 2001; Thambisetty et al., 2010), but few studies have searched for sex-related effects on brain atrophy of cardiovascular risk factors, and particularly of tobacco smoking. In addition, it should be noted that GM and WM have different relative volumes in men and women (Allen et al., 2003; Lemaître et al., 2005b; Leonard et al., 2008), which could generate discrepancies between studies having samples of different sex ratio and using WB as a phenotype of interest (see above). More generally, sex is a potential confounding factor in studies on factors of brain aging because men and women differed on many lifestyle and health parameters potentially affecting brain atrophy, and notably on tobacco smoking parameters with a larger frequency and lifetime consumption in men.

The goal of the present study was thus to take advantage of a large longitudinal community cohort study, the Three-City Study (3C Study; Alpérovitch et al., 2002), for quantifying tissue- and sex-specific effects of tobacco smoking on brain atrophy and to compare them to effects of other known factors of brain aging. An additional feature of this study is that, thanks to the longitudinal study design, we were able to estimate the tobacco smoking effects on brain atrophy both in the cross-sectional study sample and in the subsample that underwent a follow-up examination 4 years after their entry in the study. The combination of the cross-sectional and longitudinal approaches, implemented only in a few previous studies on the same topic (Enzinger et al., 2005; Durazzo et al., 2012) gave the opportunity to look at complementary aspects of tobacco smoking effects on brain atrophy, namely lifetime cumulative effects and effects on the brain of elderly individuals.

## Methods

### Population and study design

The 3C Study is a prospective cohort study, whose design has been described in detail elsewhere (Alpérovitch et al., 2002). The study took place in *three* French cities; here we use the subsample from the city of Dijon (3C-D). Briefly, 4931 non-institutionalized persons aged 65 years and over were recruited from the electoral rolls of the city of Dijon between March 1999 and March 2001. The Ethic committee of the Kremlin-Bicêtre hospital approved the 3C protocol, and all participants were asked to sign an informed consent. 3C-Dijon participants enrolled between June 1999 and September 2000, aged less than 80 years, those who were able to come to the examination center (*n* = 2763) being invited to have a brain MRI. Although 2285 persons agreed to participate, because of financial limitations, 1924 participants were scanned (3C-D-MRI subsample). As compared to participants who were not scanned, the 3C-D-MRI sample was younger (72.5 vs. 73.4 years old, *p* < 0.001), and had a lower proportion of women (62.2% vs. 71.0%, *p* < 0.001), higher education (high school graduates: 23.5% vs. 17.8%, *p* < 0.001), and better health status (62.3% vs. 56.4%, *p* < 0.001). Approximately 4 years after inclusion, 1402 of those who were scanned agreed to have a follow-up MRI (follow-up rate = 77.8%). A number of participants were later excluded due to either (1) poor technical quality of their MRI, (2) failure in MRI processing, (3) missing data (demographic, biological, cognitive, genotyping), or (4) previous history of stroke or a diagnosis of dementia according to the DSM-IV criteria (American Psychiatric Association, 1994). As a result, the sample of the present study eventually included 1451 participants at entry (920 women, 531 men) and 1111 participants at follow-up (721 women, 390 men).

### MRI acquisition and processing

MRI examinations at study entry (*t_E_*) and 4 years follow-up (*t_F_*) were acquired using the same scanner (1.5 T Siemens) and a standardized acquisition imaging protocol. High-resolution T1-weighted brain volume was acquired using a 3D inversion recovery fast spoiled-gradient echo sequence (3D SPGR; *TR* = 9.7 ms; *TE* = 4 ms; *TI* = 600 ms; coronal acquisition). The axially reoriented 3D T1 volume matrix size was 256 × 192 × 256 mm^3^, with a voxel size of 1.0 × 0.98 × 0.98 mm^3^. T2- and proton density (PD)-weighted brain volumes were acquired using a 2D fast spin echo sequence with two echo times (*TE*1 = 16 ms, *TE*2 = 98 ms, *TR* = 4400 ms). T2 and PD acquisitions consisted of 35 axial slices with 3.5 mm thickness (0.5 mm gap), 256 × 256 mm^2^ matrix size and pixel size of 0.98 × 0.98 mm^2^.

T1- and T2-weighted images of each participant were processed with SPM99, an optimized Voxel-Based Morphometry (VBM) protocol (Ashburner and Friston, 2000) that we customized in order to take into account the structural characteristics of the aged brain (Lemaître et al., 2005b). Using this VBM procedure, brain tissue probability maps were obtained for each individual and each acquisition time. We applied a modulation step to each individual's tissue probability density maps to preserve the participant original tissue quantity after being transferred to the reference space used (Good et al., 2001). At *t_E_* and *t*_F_, both gray matter (GM) white matter (WM) global volumes were estimated as the integral of voxel intensities over their modulated probability density images. At each time, whole brain (WB) volume was computed as the sum of GM and WM volumes.

For the purpose of the present study, the hippocampus was considered as a region of specific interest (ROI) given that it is a highly recognized imaging marker of brain aging (Hof and Morrison, 2004). Similar to previous studies (Lemaître et al., 2005b; Crivello et al., 2010) left and right hippocampus volumes were automatically estimated by integrating the voxel intensities of the modulated GM partition images within hippocampus limits as defined by the AAL atlas (Tzourio-Mazoyer et al., 2002). Statistical analysis was performed on the total hippocampus volume (HIP), i.e., on sum of the left and right volumes.

For the longitudinal analysis, we computed for each individual and each tissue (WB, GM, WM) and ROI (HIP), an annual rate of volume change as: △*V* = (*V_F_* − *V_E_*)/(*t_F_* − *t_E_*), *V_E_* and *V_F_* being the estimated volumes at entry and 4 year follow-up, respectively.

In this study, we did not correct the WM volume for the presence of WM lesions (WML). Rather, WML were evaluated at entry for each participant using a multi-spectral (T1, T2, PD) MRI analysis as previously reported (Maillard et al., 2008) and used, as previously done by others (Enzinger et al., 2005; Ikram et al., 2008; Durazzo et al., 2012; Hoogendam et al., 2012), as a covariate in the statistical analysis of tobacco effect on brain tissue.

### Tobacco smoking variables and other personal health-related covariates

Participant status regarding tobacco consumption was categorized as never, former and current smoker. Lifetime tobacco consumption was estimated in number of “pack-years,” i.e., the average number of packs of cigarettes smoked per day times the number of years of smoking.

Consumption of alcohol was measured used in g/day of pure alcohol. Systolic and diastolic blood pressure (SBP and DBP, respectively), fasting blood glucose and total cholesterol were measured in each individual. Hypertension was defined by systolic blood pressure (SBP) ≥140 mm Hg, or diastolic blood pressure (DBP) ≥90 mm Hg, or use of antihypertensive drugs. Diabetes mellitus was defined as fasting blood glucose ≥7 mmol/L or use of anti-diabetic drugs. Hypercholesterolemia was defined as fasting total cholesterol ≥6.2 mmol/L or use of lipid-lowering drugs. Body mass index (BMI) was calculated as the ratio of weight (kg) to the square of height (m^2^).

Educational level was defined as the number of years of scholarship since primary school. Participant's global cognitive status was evaluated using the Mini Mental State Examination (Folstein et al., 1975). Depression symptoms were evaluated using the Center for Epidemiologic Studies-Depression scale (CES-D). Genotyping of the ApoE epsilon allele polymorphism was performed as previously described (Crivello et al., 2010), participants being classified as either non-carrier or carrier of an epsilon-4 allele.

### Statistical analysis

Statistical analysis was conducted separately in men and women in order to avoid the confounding effects of sex on brain atrophy and smoking. Effects of tobacco smoking status on brain tissue volumes (WB, GM, WM, and HIP) at entry in the study or their annualized variation were studied using general linear modeling. The cross-sectional sample was used for the analysis at study entry time and the longitudinal sample for the analysis of annualized tissue volume variations. Age, educational level, MMSE score, BMI, CES-D score, SBP, DBP, fasting blood glucose, cholesterolemia, alcohol consumption, WML, and Apo-ε_4_ charge were included as covariates. Total intracranial volume (TIV) was used as a covariate for WB, GM, and WM volumes analyses, whereas GM tissue volume was used as a covariate for HIP volume analysis in order to uncover effects that might be specific to this brain area as compared to the rest of GM. Whenever a significant effect of smoking status on a volume or annualized change was found, we searched for a possible “dose-effect” through linear modeling of this tissue volume/annualized change with pack-years as the independent variable, and including the same set of covariates. For the sake of comparison with previous studies, we performed an additional analysis on the entire sample including a sex main effect and a sex by smoking interaction effect. In all statistical analyses, variables (both dependent or independent) were standardized allowing a comparison of the size of effects of the different independent variables. All statistical analyses were performed using the JMP Pro software (SAS Institute Inc., Cary, USA).

## Results

### Sample characteristics

Table 1 gives the bio-clinical characteristics of the cross-sectional and longitudinal study samples separately for men and women. At both entry and follow-up time, men and women differed on all variables except age and MMSE. Men were better educated, had higher fasting blood glucose, blood pressure, BMI, tobacco smoking and alcohol intake, and lower CES-D score and cholesterol level than women. Note, in particular, the marked difference in the frequencies of never smokers in men (30.5%) and women (82.4%).

**Table 1:** **Bio-clinical characteristics of the women and men subgroups of participants at the time of entry in the cross-sectional and longitudinal studies**.

	**Cross-sectional study sample**			**Longitudinal study sample**		
	**Men**	**Women**	***p***	**Men**	**Women**	***p***
Sample size	531	920		390	721	
Age (years)	72.3 (4.00)	72.7 (4.06)	0.097	72.0 (3.93)	72.3 (3.93)	0.27
Education (years)	9.3 (4.8)	8.2 (4.4)	<10^−4^	10.3 (4.6)	8.9 (4.2)	<10^−4^
Smoking status			<10^−4^			<10^−4^
non	162 (30.5%)	758 (82.4%)		118 (30.3%)	596 (82.7%)	
former	320 (60.3%)	128 (13.9%)		235 (60.2%)	99 (13.7%)	
current	49 (9.2%)	34 (3.7%)		37 (9.5%)	26 (3.6%)	
Alcohol intake (g/day)	20.7 (16.1)	7.37 (8.27)	<10^−4^	21.0 (16.5)	7.28 (8.23)	<10^−4^
BMI (kg.m^−2^)	25.9 (3.25)	24.9 (3.90)	<10^−4^	25.8 (3.08)	24.7 (3.72)	<10^−4^
Fasting blood glucose (mmol/L)	5.26 (1.17)	4.97 (1.14)	<10^−4^	5.29 (1.26)	4.93 (0.95)	<10^−4^
Diabetes	10.2%	6.1%	0.014	11.0%	5.8%	0.008
SBP (mm Hg)	155.8 (21.7)	144.8 (22.2)	<10^−4^	156.2 (21.4)	144.3 (22.0)	<10^−4^
DBP (mm Hg)	87.9 (11.3)	83.3 (11.4)	<10^−4^	88.3 (11.1)	83.3 (11.0)	<10^−4^
Hypertension	83.2%	72.5%	<10^−4^	83.0%	70.9%	<10^−4^
Fasting blood cholesterol (mmol/L)	5.58 (0.90)	5.91 (0.94)	<10^−4^	5.57 (0.90)	5.89 (0.93)	<10^−4^
Hypercholesterolemia	33.7%	40.9%	0.006	35.9%	41.2%	0.085
ApoE-ε_4_ genotype			0.05			0.088
non carrier	403 (76.0%)	747 (81.2%)		297 (76.2%)	587 (81.4%)	
carrier	128 (24.1%)	173 (18.8%)		93 (23.8%)	134 (18.6%)	
CES-D	7.63 (6.9)	11.7 (9.4)	<10^−4^	7.2 (6.4)	11.4 (9.2)	<10^−4^
MMSE	27.77 (1.65)	27.67 (1.81)	0.36	27.85 (1.56)	27.76 (1.76)	0.36
WML (% of WM volume)	2.25 (1.72)	2.14 (1.93)	0.30	2.21 (1.75)	2.09 (1.95)	0.32

*Values are means (standard deviations) or percentages. P is for the comparison between men and women (Student's t-tests or Chi-squared tests). BMI, body mass index; SBP and DBP, systolic and diastolic blood pressures; CES-D, Center for epidemiological study depression scale; MMSE, mini-mental scale examination. WML, white matter lesion as a percentage of white matter (WM) volume*.

Participants of the cross-sectional sample who did not have a follow-up differed from those of the longitudinal sample on several parameters: they were older at their entry in the study (73.2 vs. 72.0 years, *p* = 0.004 for men and 74.1 vs. 72.3 years, *p* < 10^−4^ for women), had a lower education level (6.6 vs. 10.3 years in men and 5.6 vs. 8.9 years in women, *p* < 10^−4^ in both sexes), larger CES-D scores (8.9 vs. 7.1, *p* = 0.009 in men and 13.0 vs. 11.3, *p* = 0.026 in women) and lower MMSE (27.5 vs. 27.8, *p* = 0.042 in men and 27.3 vs. 27.7, *p* = 0.006 in women). Moreover in women they also differed for BMI (25.6 vs. 24.7 kg/m^2^, *p* = 0.005) and glycaemia (5.1 vs. 4.9 mmol/l, *p* = 0.039).

Table 2 describes the smoking history parameters of male and female former and current smokers, in the cross-sectional and longitudinal samples. Besides the above-mentioned difference in smoking status frequencies, men and women also differed regarding the age at which they started smoking (men starting 4 years earlier than women, *p* < 10^−4^ for former smokers and *p* = 0.0037 for current smokers). Regarding former smokers, men smoked 3 years more than women, *p* = 0.009) and had a higher lifetime tobacco consumption, consuming 6 pack-years more than women (*p* = 0.0037). Such differences between men and women were not observed in current smokers (*p* = 0.27 and *p* = 0.14, respectively). Note that the most important differences were between the former and current smoker groups in both sexes, both in term of number of years of smoking (*p* < 10^−4^ for both sexes) and number of pack-years (*p* < 10^−4^ for men and *p* = 0.0019 in women).

**Table 2:** **Smoking history of former and current smokers in the baseline and follow-up samples**.

	**Cross-sectional study sample**				**Longitudinal study sample**			
	**Men**		**Women**		**Men**		**Women**	
	**Former**	**Current**	**Former**	**Current**	**Former**	**Current**	**Former**	**Current**
Age when smoking start	18.9 (4.0)	19.2 (5.3)	23.2 (7.4)	24.1 (9.5)	18.7 (4.0)	19.4 (5.9)	23.2 (7.6)	21.6 (7.8)
Age when smoking quit	46.3 (13.0)	n.a.	46.9 (13.6)	n.a.	46.1 (12.5)	n.a.	47.3 (14.1)	n.a.
Years of smoking	27.4 (13.3)	51.1 (12.5)	23.7 (14.0)	48.2 (10.4)	27.3 (12.7)	52.4 (7.0)	24.1 (14.2)	50.6 (8.9)
Years free of smoking	25.9 (12.5)	n.a.	25.1 (13.8)	n.a.	26.0 (12.4)	n.a.	24.5 (13.8)	n.a.
Smoked pack-years	21.4 (18.4)	35.6 (20.2)	15.2 (23.5)	29.0 (18.8)	21.5 (12.5)	33.5 (21.7)	15.3 (24.3)	29.3 (17.3)

*Values are means (standard deviations) at the time of participant entry in the study. “Pack-years” are the number of packs of cigarettes smoked per day times the number of years of smoking*.

Average brain phenotypes derived from MRI are shown in Table 3. At entry, cross-sectional and longitudinal samples slightly differed regarding HIP volume in men (*p* = 0.001), and WB (*p* = 0.022), GM (*p* = 0.0007) and HIP (*p* < 10^−4^) volumes in women. As expected, men exhibited larger TIV, WB, GM, WM, and HIP volumes than women both at entry and follow-up (*p* < 10^−4^, in all cases). Regarding annualized variation of these phenotypes, we found significant loss of WB, GM, WM, and HIP volumes in both men and women (*p* < 10^−4^, in all cases). Note that, although men and women did not significantly differ in their annualized rate of WB loss, women exhibited significantly larger annual loss of GM than men, while the opposite was found for WM. However, there was no difference between men and women in their annual rate of HIP volume loss.

**Table 3:** **MRI-derived brain phenotypes of the men and women subgroups of participants at entry and follow-up (about 4 years after their entry in the study), and their annual variation**.

	**Cross-sectional sample**		**Longitudinal sample**		**Longitudinal sample**		**Longitudinal sample**		
	**At entry**		**At entry**		**At follow-up**		**Annual variation**		
	**Men**	**Women**	**Men**	**Women**	**Men**	**Women**	**Men**	**Women**	***p***
TIV	1462 (116)	1297 (94)	1462 (111)	1294 (94)	1460 (111)	1291 (94)	−0.48 (1.25)	−0.73 (1.03)	0.0003
nS	1484 (122)	1297 (98)	1491 (114)	1293 (93)	1489 (114)	1290 (93)	−0.56 (1.08)	−0.76 (1.05)	0.061
fS	1450 (114)	1293 (102)	1446 (110)	1290 (96)	1445 (111)	1288 (96)	−0.41 (1.35)	−0.58 (0.91)	0.27
cS	1463 (100)	1317 (125)	1471 (91)	1330 (118)	1469 (90)	1328 (118)	−0.61 (1.15)	−0.59 (0.90)	0.95
WB	1043 (86)	937 (76)	1046 (88)	940 (76)	1028 (86)	921 (73)	−5.00 (3.06)	−5.34 (2.92)	0.068
nS	1062 (89)	937 (75)	1070 (91)	940 (74)	1052 (90)	921 (72)	−5.13 (2.73)	−5.38 (2.89)	0.37
fS	1036 (86)	933 (78)	1033 (87)	935 (75)	1016 (86)	918 (72)	−4.73 (2.95)	−4.88 (3.05)	0.67
cS	1032 (67)	942 (86)	1045 (65)	952 (91)	1023 (61)	930 (93)	−6.32 (4.24)	−6.14 (3.02)	0.86
GM	539 (46)	489 (42)	540 (47)	492 (42)	527 (45)	476 (40)	−3.63 (3.27)	−4.49 (2.98)	<10^−4^
nS	548 (50)	489 (41)	553 (50)	492 (41)	539 (50)	475 (40)	−3.81 (3.06)	−4.52 (2.94)	0.018
fS	535 (45)	490 (46)	535 (45)	492 (45)	522 (44)	477 (41)	−3.43 (3.25)	−4.19 (3.27)	0.054
cS	531 (36)	495 (50)	535 (37)	501 (54)	519 (33)	483 (55)	−4.33 (3.92)	−4.94 (2.78)	0.50
WM	505 (48)	447 (42)	506 (49)	447 (42)	500 (48)	445 (41)	−1.37 (2.26)	−0.85 (2.20)	0.0002
nS	514 (47)	448 (43)	517 (48)	449 (42)	513 (49)	446 (41)	−1.31 (2.33)	−0.86 (2.21)	0.044
fS	501 (49)	443 (40)	499 (49)	443 (38)	494 (49)	441 (39)	−1.30 (2.21)	−0.70 (2.21)	0.024
cS	502 (41)	446 (44)	510 (39)	451 (46)	504 (38)	447 (49)	−1.99 (2.27)	−1.21 (1.85)	0.15
HIP	7.01 (0.84)	6.40 (0.73)	7.08 (0.83)	6.46 (0.72)	6.84 (0.87)	6.20 (0.78)	−0.066 (0.069)	−0.071 (0.062)	0.17
nS	7.11 (0.85)	6.41 (0.73)	7.21 (0.84)	6.46 (0.72)	6.97 (0.88)	6.22 (0.78)	−0.069 (0.063)	−0.069 (0.061)	0.92
fS	6.97 (0.84)	6.32 (0.71)	7.03 (0.84)	6.39 (0.71)	6.82 (0.88)	6.12 (0.74)	−0.061 (0.067)	−0.074 (0.062)	0.090
cS	6.88 (0.63)	6.44 (0.76)	6.92 (0.63)	6.52 (0.80)	6.61 (0.70)	6.14 (0.87)	−0.089 (0.088)	−0.11 (0.071)	0.41

*Values are means (standard deviations, in cm^3^) for the cross-sectional study sample at entry, and for the longitudinal study sample at entry and at 4-year follow-up. TIV, total intracranial volume; WB, whole brain volume (GM + WM); GM, gray matter volume; WM, white matter volume; HIP, hippocampus volume (left plus right). Annual variation (in cm^3^/year) is the ratio of the difference between follow-up and entry volume values to the delay between follow-up and entry. nS, non-smokers; fS, former smokers; cS, current smokers; p is for the comparison of annual variation of volumes between men and women (One-Way ANOVA)*.

### Effect of tobacco smoking on brain tissue volumes

#### Whole brain

**Figure 1:**
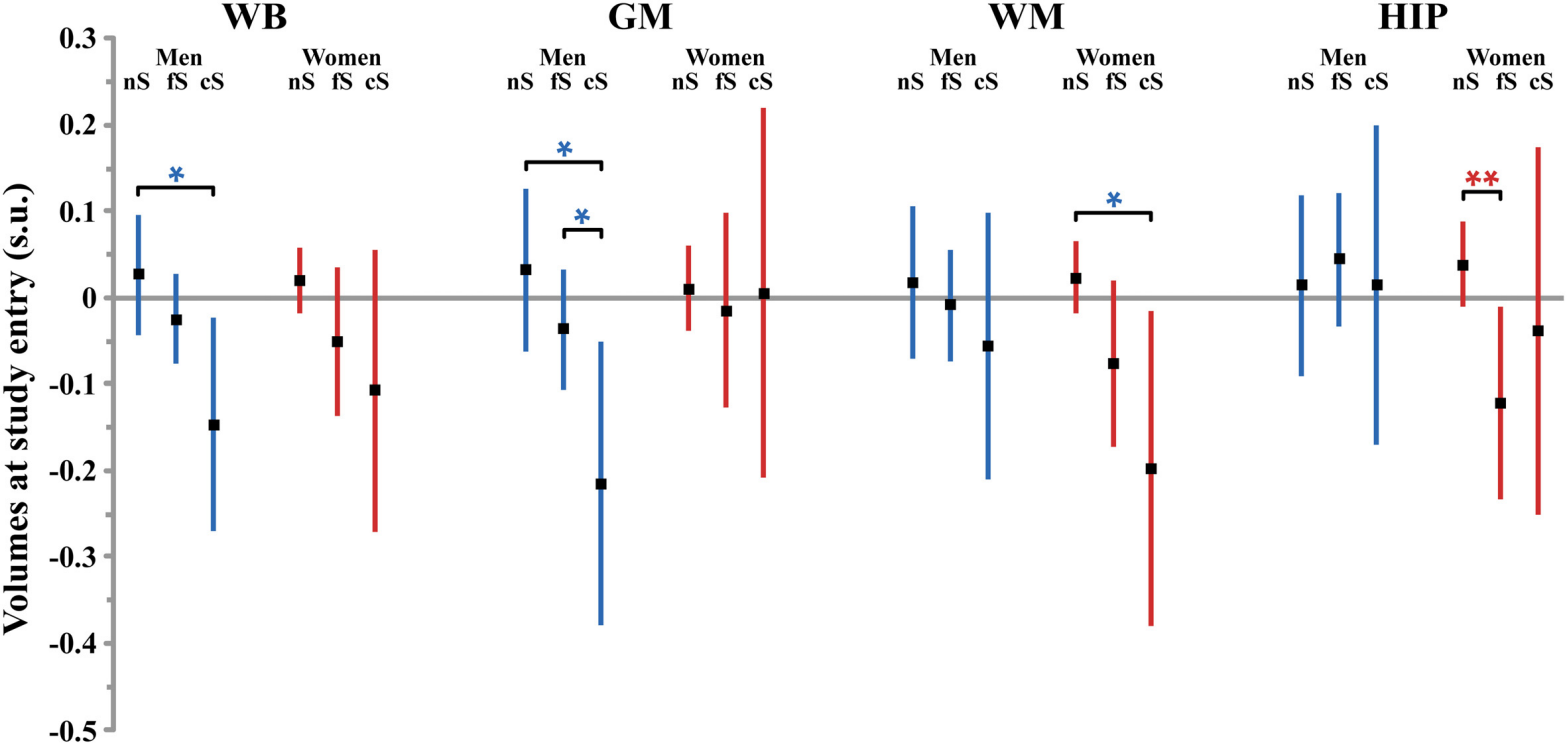
**Size (and 95% confidence interval, in standardized unit (s.u.) of the effects of smoking on cerebral phenotypes volumes at entry in men (blue bars) and women (red bars)**. WB, whole brain volume (GM + WM); GM, gray matter volume; WM, white matter volume; HIP, hippocampus volume (left plus right). nS, non-smoker; fS, former smoker; cS, current smokers. Significance level of *post-hoc t*-tests for the comparison between subgroups of differing smoking status ^*^p < 0.05; ^**^p < 0.01; ^†^*p* < 0.001; ^‡^*p* < 0.0001.

Table 4 reports the effects of tobacco smoking on WB volume, effects size and *post-hoc* significance reported in Figure 1. Effect on WB volume at entry failed to reach significance both in men and in women (*p* = 0.057 and *p* = 0.12, respectively). The trend observed in men was due to a lower WB volume in current smokers compared to never smokers (*p* = 0.017, *post-hoc* test, see Figure 1). There was no significant linear correlation between WB volume and pack-years of men current smokers (regression slope = −0.027, *p* = 0.64, *N* = 49).

**Table 4:** **Cross-sectional and longitudinal analyses of tobacco smoking and other covariate effects on whole brain volume**.

	**Cross-sectional analysis (entry time)**				**Longitudinal analysis**			
	**Men**		**Women**		**Men**		**Women**	
	**Effect size**	***p*-value**	**Effect size**	***p*-value**	**Effect size**	***p*-value**	**Effect size**	***p*-value**
Smoking status	^*^	0.057	^*^	0.12	^*^	**0.034**	^*^	0.12
Age	**−0.19**	**<10^−4^**	**−0.26**	**<10^−4^**	−0.016	0.77	**0.098**	**0.011**
ApoE-_ε4_^†^	0.081	0.061	−0.012	0.77	−0.10	0.39	−0.084	0.37
Alcohol	**−0.043**	**0.025**	−0.025	0.12	0.023	0.66	**0.076**	**0.041**
BMI	0.011	0.58	0.023	0.18	0.045	0.40	0.0034	0.93
CES-D	0.032	0.088	−0.023	0.15	0.047	0.35	0.013	0.73
Cholesterolemia	0.0087	0.64	−0.030	0.065	−0.083	0.10	0.031	0.41
Education	**−0.070**	**0.0004**	**−0.036**	**0**.**038**	−0.038	0.46	0.049	0.21
Glycaemia	**−0.077**	**<10^−4^**	−0.023	0.17	−0.029	0.57	**−0.10**	**0.009**
MMSE	−0.0024	0.90	−0.0031	0.86	−0.083	0.12	−0.00026	0.99
SBP	−0.011	0.66	0.035	0.11	−0.0059	0.93	−0.076	0.14
DBP	0.0083	0.74	−0.018	0.41	−0.0031	0.97	0.082	0.10
WML	−0.015	0.42	−0.010	0.54	0.0073	0.88	0.040	0.28
TIV	**0.86**	**<10^−4^**	**0.83**	**<10^−4^**	**−0.17**	**0.0011**	**−0.13**	**0.0007**

Effect size is in standardized unit. P-value is the effect significance level. BMI, body mass index; SBP and DBP, systolic and diastolic blood pressure; CES-D, Center for epidemiological study depression scale; MMSE, mini-mental scale examination; WML, white matter lesion. TIV, total intracranial volume.

* See Figures 1, 2;

† Effect size is carriers–non carriers. Bold values indicate effect significance level < 0.05.

**Figure 2:**
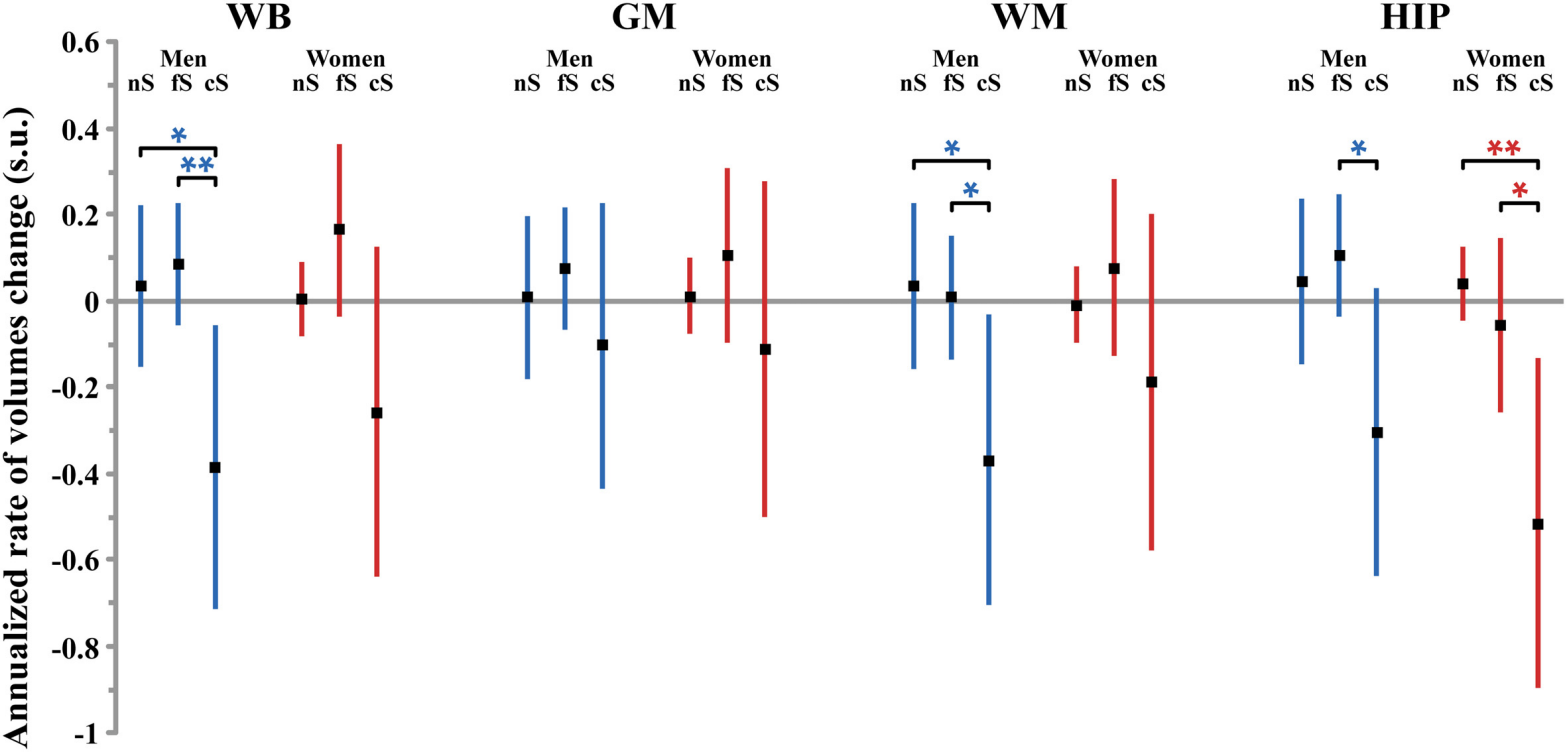
**Size (and 95% confidence interval, in standardized unit (s.u.) of the effects of smoking on cerebral phenotype annualized rates of change in men (blue bars) and women (red bars)**. WB, whole brain volume (GM + WM); GM, gray matter volume; WM, white matter volume; HIP, hippocampus volume (left plus right). nS, non-smoker; fS, former smoker; cS, current smokers. Significance level of *post-hoc t*-tests for the comparison between subgroups of differing smoking status ^*^p < 0.05; ^**^p < 0.01; ^†^*p* < 0.001; ^‡^*p* < 0.0001.

The annualized rate of WB loss was significantly affected by smoking status in men but not in women (*p* = 0.034 and *p* = 0.12, respectively), although both groups exhibited similar profiles (see Figure 2). In men, current smokers exhibited significantly higher WB rate of atrophy than both never smokers and former smokers (*p* = 0.0094 and *p* = 0.031, respectively; *post-hoc t*-test). However, there was no significant linear correlation between WB rate of atrophy and pack-years in men current smokers (*p* = 0.10).

#### Gray matter

Here, the patterns of smoking were very different between men and women. Smoking was found to have a significant effect on GM volume at entry in men but not in women (*p* = 0.039 and *p* = 0.92, respectively, Table 5). Men who were current smokers at the time of the study had a smaller GM volume than both never smokers and former smokers (*p* = 0.011 and *p* = 0.047, respectively, *post-hoc t*-tests, Figure 1), while there here was no difference between the two latter groups (*p* = 0.23, *post-hoc t*-test). The size of the effect of smoking in men (0.25 s.u. for the difference between never smokers and current smokers) was similar to that of age (0.25 s.u.) and much larger than those of the other covariates (except TIV). Such reduction of GM volume in current smokers was not significantly correlated with their pack-years (*p* = 0.64).

**Table 5:** **Cross-sectional and longitudinal analyses of tobacco smoking and other covariate effects on gray matter volume**.

	**Cross-sectional analysis (entry time)**				**Longitudinal analysis**			
	**Men**		**Women**		**Men**		**Women**	
	**Effect size**	***p*-value**	**Effect size**	***p*-value**	**Effect size**	***p*-value**	**Effect size**	***p*-value**
Smoking status	^*^	**0.039**	^*^	0.92	^*^	0.57	^*^	0.55
Age	**−0.25**	**<10^−4^**	**−0.35**	**<10^−4^**	−0.062	0.25	0.072	0.066
ApoE-_ε4_^†^	**0.13**	**0.025**	−0.028	0.60	−0.15	0.20	−0.10	0.29
Alcohol	−0.0078	0.76	−0.030	0.15	−0.053	0.30	**0.079**	**0.036**
BMI	−0.013	0.62	−0.0072	0.74	0.0065	0.90	0.039	0.33
CES-D	0.00059	0.98	−0.037	0.083	0.097	0.059	0.028	0.46
Cholesterolemia	−0.0079	0.75	−0.020	0.34	−0.083	0.10	0.062	0.10
Education	−0.031	0.23	0.015	0.50	−0.020	0.70	0.020	0.61
Glycaemia	**−0.076**	**0.003**	−0.028	0.20	−0.032	0.53	**−0.079**	**0.043**
MMSE	0.033	0.21	0.025	0.26	−0.087	0.10	−0.0037	0.92
SBP	−0.015	0.65	0.043	0.14	0.025	0.73	−0.034	0.52
DBP	−0.010	0.76	−0.032	0.26	−0.049	0.49	0.028	0.58
WML	**−0.092**	**0.0003**	**−0.090**	**<10^−4^**	−0.034	0.51	0.00072	0.98
TIV	**0.74**	**<10^−4^**	**0.65**	**<10^−4^**	**−0.18**	**0.001**	**−0.075**	**0.047**

Effect size is in standardized unit. P-value is the effect significance level. BMI, body mass index; SBP and DBP, systolic and diastolic blood pressure; CES-D, Center for epidemiological study depression scale; MMSE, mini-mental scale examination. WML, white matter lesion. TIV, total intracranial volume.

* See Figures 1, 2;

† Effect size is carriers–non carriers. Bold values indicate effect significance level < 0.05.

Meanwhile, we found that the annualized rate of loss of GM was not modified by the participant smoking status, neither in men (*p* = 0.57) nor in women (*p* = 0.55).

#### White matter

Smoking was found to have a significant effect on WM volume at entry in women but not in men (*p* = 0.015 and *p* = 0.71, respectively, Table 6). Women who were never smokers had a significantly larger WM volume than current smokers (*p* = 0.020, *post-hoc t*-test, Figure 1) while the difference with the former smokers was close to significance (*p* = 0.055, *post-hoc t*-test). In addition, the linear correlation between pack-years and WM volume at entry time in women who were either former or current smokers was close to significance (regression slope = −0.077, *p* = 0.062).

**Table 6:** **Cross-sectional and longitudinal analyses of tobacco smoking and other covariate effects on white matter volume**.

	**Cross-sectional analysis (entry time)**				**Longitudinal analysis**			
	**Men**		**Women**		**Men**		**Women**	
	**Effect size**	***p*-value**	**Effect size**	***p*-value**	**Effect size**	***p*-value**	**Effect size**	***p*-value**
Smoking status	^*^	0.71	^*^	**0.015**	^*^	0.10	^*^	0.47
Age	**−0.11**	**<10^−4^**	**−0.11**	**<10^−4^**	0.068	0.21	0.033	0.41
ApoE-_ε4_^†^	0.020	0.71	0.0060	0.90	0.084	0.48	0.026	0.79
Alcohol	**−0.069**	**0.004**	−0.014	0.43	**0.11**	**0.041**	−0.0060	0.87
BMI	0.032	0.19	**0.048**	**0.011**	0.052	0.35	−0.048	0.23
CES-D	**0.057**	**0.016**	−0.0048	0.79	−0.076	0.15	−0.021	0.59
Cholesterolemia	0.023	0.32	−0.033	0.066	0.0084	0.87	−0.043	0.25
Education	**−0.095**	**<10^−4^**	**−0.079**	**<10^−4^**	−0.023	0.67	0.038	0.34
Glycaemia	**−0.065**	**0.007**	−0.013	0.48	0.0070	0.89	−0.027	0.50
MMSE	−0.036	0.14	−0.030	0.11	0.014	0.80	0.0047	0.90
SBP	−0.0050	0.88	0.020	0.42	−0.044	0.55	−0.055	0.29
DBP	0.025	0.43	−0.00028	0.99	0.068	0.35	0.070	0.17
WML	**0.061**	**<10^−4^**	**0.071**	**<10^−4^**	0.059	0.26	0.053	0.17
TIV	**0.82**	**0.010**	**0.85**	**<10^−4^**	0.019	0.72	−0.067	0.081

Effect size is in standardized unit. P-value is the effect significance level. BMI, body mass index; SBP and DBP, systolic and diastolic blood pressure; CES-D, Center for epidemiological study depression scale; MMSE, mini-mental scale examination. WML, white matter lesion. TIV, total intracranial volume.

* See Figures 1, 2;

† Effect size is carriers–non carriers. Bold values indicate effect significance level < 0.05.

There was no effect of smoking on the annualized loss of WM tissue, neither in men (*p* = 0.10) nor in women (*p* = 0.47).

#### Hippocampus

**Figure 3:**
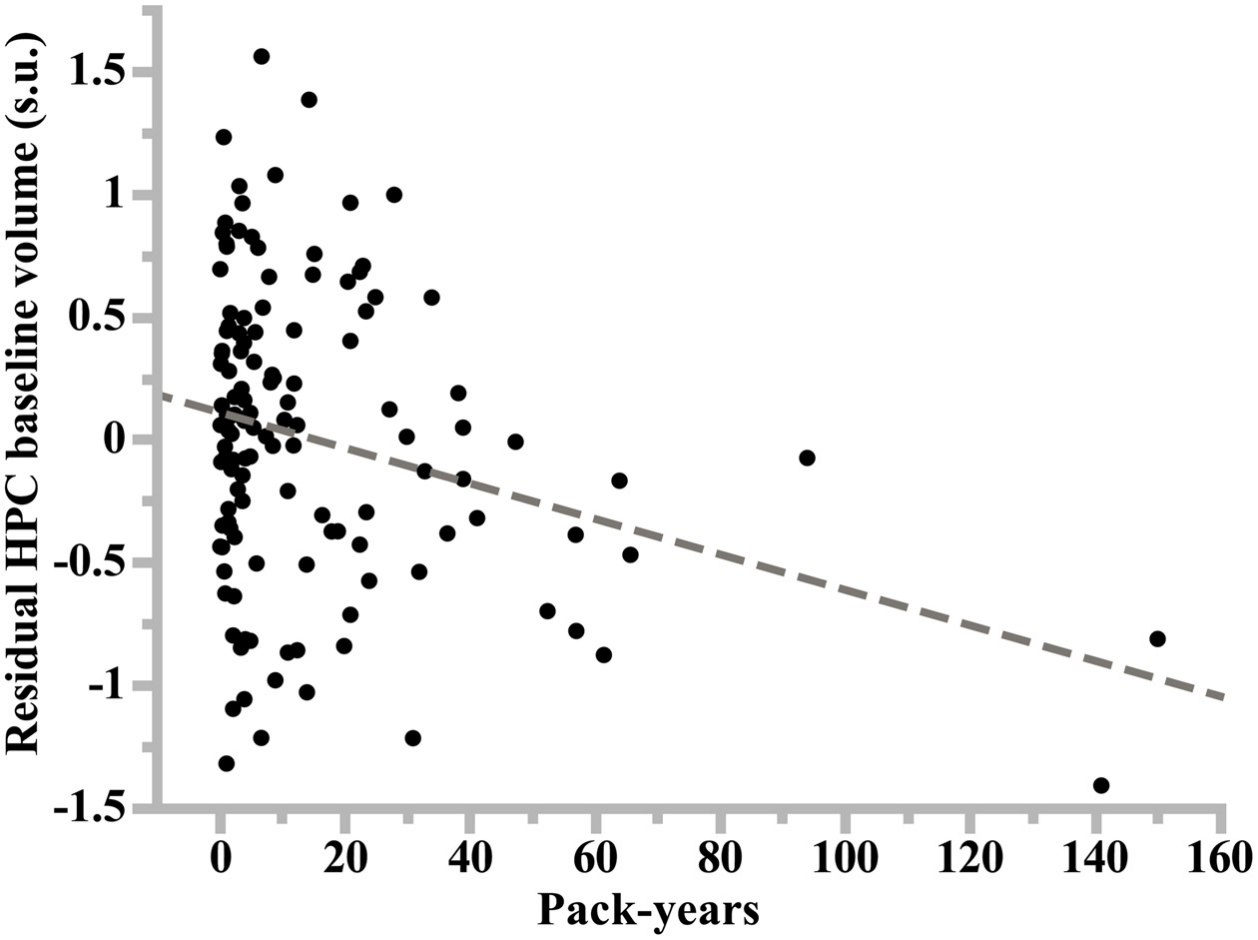
**Plot of the linear regression analysis of hippocampus volume at study entry (residual values in standardized unit) as a function of lifetime cumulative cigarette smoking (in pack-years: number of packs smoked per day times number of years of smoking) in former smoker women**. Slope of the regression line: −0.17, *p* = 10^−4^, *N* = 128.

Smoking was found to have additional significant specific effects on hippocampal volume in women but not in men (*p* = 0.028 and *p* = 0.89, respectively; Table 7). Specifically, former smoker women had smaller HIP volume than never smokers (*p* = 0.008, *post-hoc t*-test; see Figure 1), while there was no difference between current smokers and the two other subgroups (*p* = 0.56 and *p* = 0.43 for the comparison with non-smokers and former smokers, respectively, *post-hoc t*-test). In addition, a very significant linear correlation was found between pack-years and HIP volume at entry time in women who were former smokers (regression slope = –0.17, *p* = 0.0014, *N* = 128, see Figure 3).

**Table 7:** **Cross-sectional and longitudinal analyses of tobacco smoking and other covariate effects on hippocampus volume**.

	**Cross-sectional analysis (entry time)**				**Longitudinal analysis**			
	**Men**		**Women**		**Men**		**Women**	
	**Effect size**	***p*-value**	**Effect size**	***p*-value**	**Effect size**	***p*-value**	**Effect size**	***p*-value**
Smoking status	^*^	0.89	^*^	**0.028**	^*^	0.080	^*^	**0.016**
Age	**−0.12**	**0.0001**	**−0.15**	**<10^−4^**	0.081	0.15	**−0.11**	**0.009**
ApoE-_ε4_^†^	**−0.13**	**0.047**	−0.069	0.20	−0.20	0.094	−0.035	0.71
Alcohol	−0.055	0.054	**0.043**	**0.039**	0.00001	0.99	0.051	0.17
BMI	0.049	0.098	0.022	0.31	0.067	0.21	−0.023	0.56
CES-D	−0.052	0.065	**−0.045**	**0.030**	0.028	0.59	−0.035	0.35
Cholesterolemia	−0.025	0.38	−0.025	0.22	−0.057	0.27	0.029	0.44
Education	0.029	0.31	−0.031	0.17	−0.025	0.63	−0.0070	0.86
Glycaemia	−0.042	0.14	−0.019	0.36	0.029	0.58	0.064	0.097
MMSE	−0.032	0.28	−0.00063	0.98	−0.037	0.49	−0.014	0.72
SBP	0.030	0.44	0.026	0.36	−0.044	0.55	0.067	0.19
DBP	−0.0085	0.82	−0.034	0.24	0.065	0.37	0.00065	0.99
WML	−0.043	0.13	**−0.045**	**0.033**	−0.043	0.40	**−0.14**	**0.0003**
GM	**0.72**	**<10^−4^**	**0.71**	**<10^−4^**	−0.11	0.055	−0.0085	0.84

Effect size is in standardized unit. P-value is the effect significance level. BMI, body mass index; SBP and DBP, systolic and diastolic blood pressure; CES-D, Center for epidemiological study depression scale; MMSE, mini-mental scale examination. WML, white matter lesion. GM, gray matter volume.

* See Figures 1, 2;

† Effect size is carriers–non carriers. Bold values indicate effect significance level < 0.05.

Regarding the annualized rate of HIP volume loss (corrected for whole GM volume at entry), we found an effect of smoking that was significant in women (*p* = 0.016) and close to significance in men (*p* = 0.08). *Post-hoc t*-tests revealed the same pattern for men and women (see Figure 2), namely that the HIP volume annualized loss was larger for current smokers than either for never smokers (*p* = 0.078 and *p* = 0.0053, for men and women, respectively, *post-hoc t*-tests) or for former smokers (*p* = 0.025 and *p* = 0.036, for men and women, respectively, *post-hoc t*-tests). Such a larger rate of HIP volume loss was not correlated with the current smokers pack-years, either in men (*p* = 0.09) or in women (*p* = 0.69).

#### Pooled analysis

Analysis of baseline volumes in the pooled sample of men and women (see Table 8) revealed a significant effect of smoking on WB and WM, but not on GM and HIP, current smokers having significant lower WB and WM volumes than non-smokers (volume difference = 0.13 s.u. in both cases, *p* = 0.004 and *p* = 0.023, for WB and WM, respectively), and former smokers having lower WB volume than non-smokers (volume difference = 0.053 s.u., *p* = 0.046). There was a sex main effect on WB and GM volumes, women having smaller TIV-corrected WB and GM volumes than men (WB volume difference = 0.081 s.u., *p* = 0.043; GM volume difference = 0.12 s.u., *p* = 0.031). There was no sex by smoking interaction on any baseline volume.

**Table 8:** **Cross-sectional analysis of tobacco smoking and other covariate effects on tissue volumes in the pooled sample of men and women**.

	**WB**		**GM**		**WM**		**HIP**	
	**Effect size**	***p*-value**	**Effect size**	***p*-value**	**Effect size**	***p*-value**	**Effect size**	***p*-value**
Smoking status		**0.007**		0.11		**0.039**		0.31
Sex ^*^	**0.081**	**0.043**	**0.12**	**0.031**	0.034	0.47	−0.052	0.38
Smoking by sex		0.74		0.16		0.55		0.12
Age	**−0.19**	**<10^−4^**	**−0.28**	**<10^−4^**	**−0.089**	**<10^−4^**	**−0.12**	**<10^−4^**
ApoE-_ε4_^†^	0.027	0.28	0.038	0.27	0.012	0.68	**−0.095**	**0.013**
Alcohol	**−0.036**	**0.002**	−0.022	0.17	**−0.044**	**0.002**	−0.012	0.48
BMI	0.017	0.12	−0.0071	0.64	**0.037**	**0.004**	0.029	0.084
CES-D	−0.0058	0.58	−0.024	0.095	0.012	0.32	**−0.044**	**0.006**
Cholesterolemia	−0.012	0.26	−0.012	0.40	−0.0097	0.43	−0.022	0.16
Education	**−0.042**	**<10^−4^**	−0.0033	0.83	**−0.073**	**<10^−4^**	−0.0042	0.80
Glycaemia	**−0.038**	**0.0004**	**−0.041**	**0.005**	**−0.030**	**0.018**	−0.029	0.073
MMSE	−0.0013	0.91	0.024	0.10	**−0.025**	**0.048**	−0.011	0.51
SBP	0.015	0.30	0.019	0.33	0.0088	0.61	0.023	0.30
DBP	−0.0075	0.60	−0.023	0.23	0.0086	0.61	−0.019	0.38
WML	−0.0091	0.38	**−0.078**	**<10^−4^**	**0.057**	**<10^−4^**	**−0.041**	**0.009**
TIV (^‡^SG)	**0.89**	**<10^−4^**	**0.75**	**<10^−4^**	**0.89**	**<10^−4^**	**0.75^‡^**	**<10^−4^**

WB, whole brain volume (GM + WM); GM, gray matter volume; WM, white matter volume; HIP, hippocampus volume (left plus right). Effect size is in standardized unit. P-value is the effect significance level. BMI, body mass index; SBP and DBP, systolic and diastolic blood pressure; CES-D, Center for epidemiological study depression scale; MMSE, mini-mental scale examination. WML, white matter lesion. TIV, total intracranial volume.

* Effect size is men–women;

† Effect size is carriers–non carriers.

‡ *SG used as a covariate instead of TIV. Bold values indicate effect significance level < 0.05*.

Regarding annualized rate of tissue volume losses (Table 9), we found a significant effect of smoking for WB and HIP only: in both cases, current smokers had increased rates of tissue loss when compared to either never or former smokers (difference in rates of WB volume loss = 0.34 and 0.45 s.u., respectively, *p* = 0.014 and 0.001, respectively; difference in rates of HIP volume loss = 0.45 s.u. in both cases, *p* = 0.001 in both cases). There was a sex main effect on the annualized WB and GM volume loss, women having larger rates of volume loss than men (difference in rates of volume loss = 0.34 and 0.48 s.u., *p* = 0.005 and *p* < 10^−4^, respectively). There was no sex by smoking interaction on any annualized tissue volume loss.

**Table 9:** **Longitudinal analysis of tobacco smoking and other covariate effects on the annualized rate of tissue volume losses in the pooled sample of men and women**.

	**WB**		**GM**		**WM**		**HIP**	
	**Effect size**	***p*-value**	**Effect size**	***p*-value**	**Effect size**	***p*-value**	**Effect size**	***p*-value**
Smoking status		**0.005**		0.23		0.12		**0.004**
Sex ^*^	**0.34**	**0.005**	**0.48**	**<10^−4^**	−0.22	0.079	0.015	0.90
Smoking by sex		0.68		0.93		0.78		0.47
Age	**0.064**	**0.039**	0.034	0.28	0.038	0.22	**−0.097**	**0.004**
ApoE-_ε4_^†^	−0.11	0.15	−0.14	0.065	0.049	0.51	−0.11	0.15
Alcohol	0.066	0.055	0.020	0.55	0.059	0.088	0.024	0.48
BMI	0.010	0.75	0.022	0.49	−0.017	0.59	0.0092	0.77
CES-D	0.021	0.50	0.046	0.14	−0.036	0.25	−0.020	0.52
Cholesterolemia	−0.0060	0.84	0.011	0.71	−0.024	0.44	−0.0069	0.82
Education	0.015	0.63	0.0049	0.88	0.013	0.68	−0.012	0.71
Glycaemia	**−0.066**	**0.034**	−0.054	0.082	−0.012	0.70	0.053	0.094
MMSE	−0.026	0.41	−0.027	0.38	0.0041	0.90	−0.022	0.49
SBP	−0.060	0.16	−0.020	0.64	−0.052	0.23	0.031	0.47
DBP	0.057	0.17	0.0060	0.89	0.068	0.11	0.019	0.64
WML	0.032	0.29	−0.0077	0.80	0.053	0.083	**−0.10**	**0.0006**
TIV (^‡^SG)	**−0.18**	**<10^−4^**	**−0.14**	**0.0002**	−0.044	0.26	−0.056^‡^	0.14

WB, whole brain volume (GM + WM); GM, gray matter volume; WM, white matter volume; HIP, hippocampus volume (left plus right). Effect size is in standardized unit. P-value is the effect significance level. BMI, body mass index; SBP and DBP, systolic and diastolic blood pressure; CES-D, Center for epidemiological study depression scale; MMSE, mini-mental scale examination. WML, white matter lesion. TIV, total intracranial volume.

* Effect size is men—women;

† Effect size is carriers–non carriers.

‡ *SG used as a covariate instead of TIV. Bold values indicate effect significance level < 0.05*.

#### Effects of covariates

**Figure 4:**
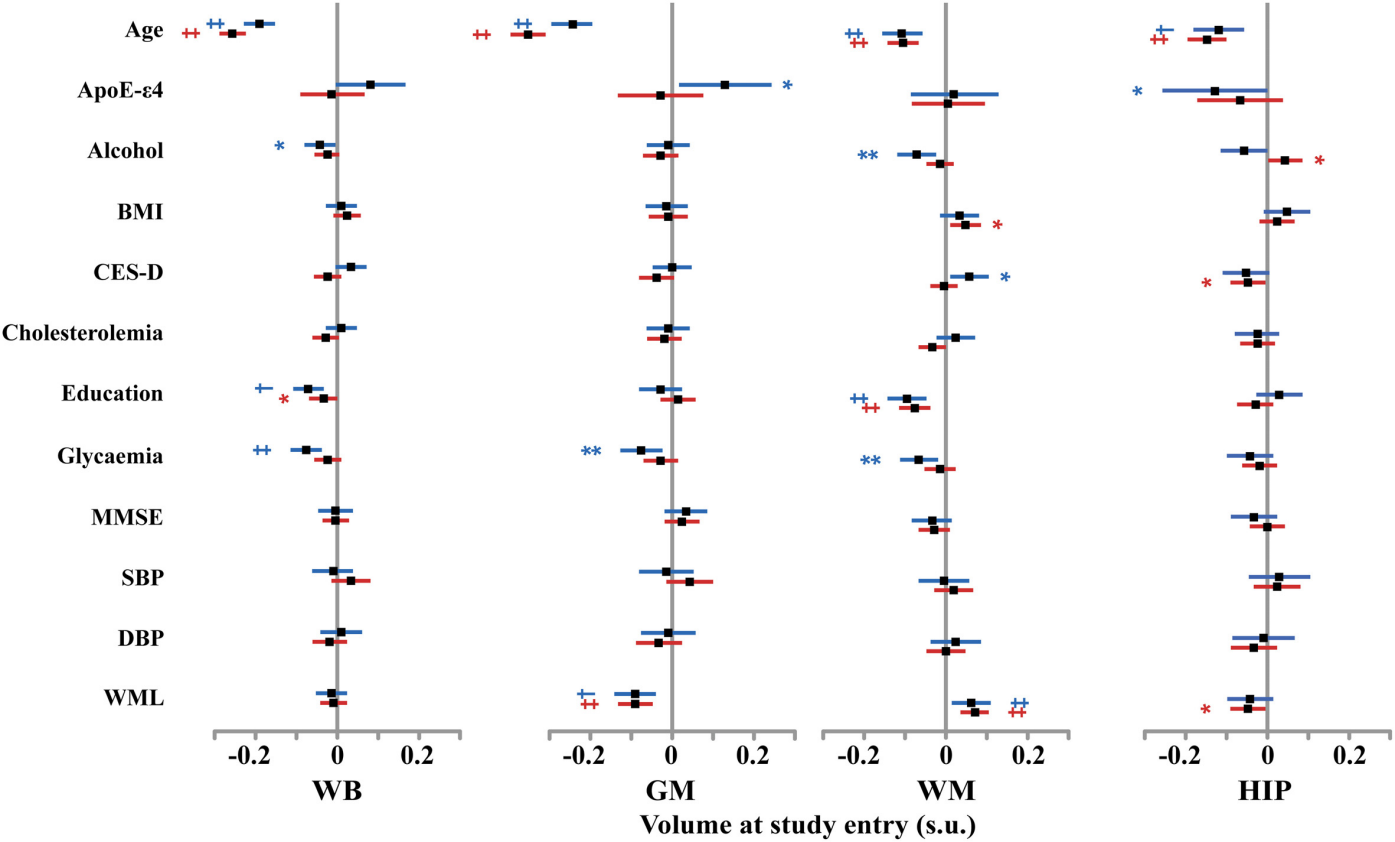
**Size and 95% confidence interval, in standardized unit (s.u.) of the effects of various covariates on cerebral phenotype volumes at entry in men (blue bars) and women (red bars)**. WB, whole brain volume (GM + WM); GM, gray matter volume; WM, white matter volume; HIP, hippocampus volume (left plus right). BMI, body mass index; SBP and DBP, systolic and diastolic blood pressure; CES-D, Center for epidemiological study depression scale; MMSE, mini-mental scale examination. WML, white matter lesion. TIV, total intracranial volume. Significance level of *post-hoc t*-tests for the comparison between subgroups of differing smoking status ^*^p < 0.05; ^**^p < 0.01; ^†^*p* < 0.001; ^‡^*p* < 0.0001.

**Figure 5:**
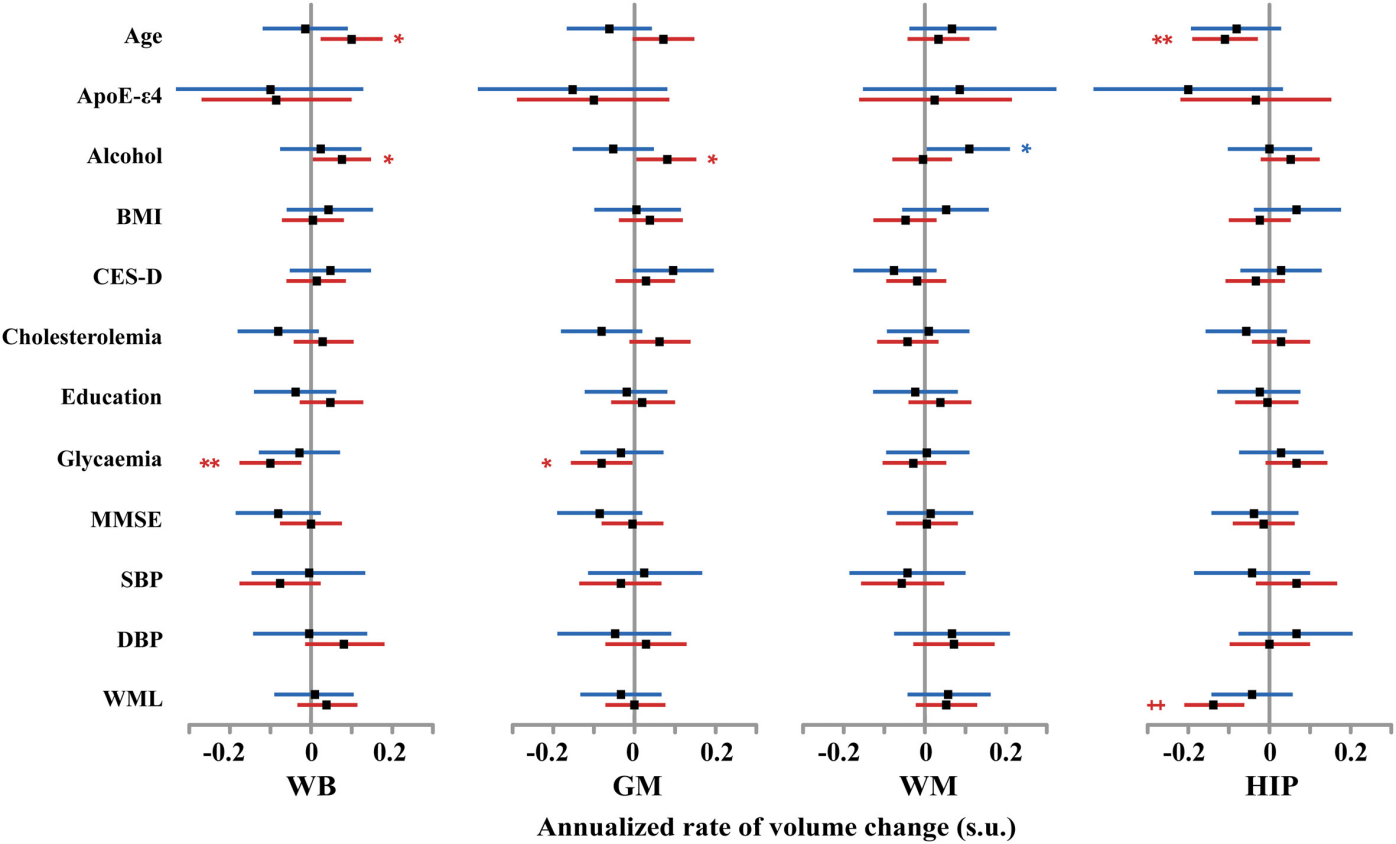
**Size and 95% confidence interval, in standardized unit (s.u.) of the effects of various covariates on cerebral phenotype annualized rates of change in men (blue bars) and women (red bars)**. WB, whole brain volume (GM + WM); GM, gray matter volume; WM, white matter volume; HIP, hippocampus volume (left plus right). BMI, body mass index; SBP and DBP, systolic and diastolic blood pressure; CES-D, Center for epidemiological study depression scale; MMSE, mini-mental scale examination. WML, white matter lesion. TIV, total intracranial volume. Significance level of *post-hoc t*-tests for the comparison between subgroups of differing smoking status ^*^p < 0.05; ^**^p < 0.01; ^†^*p* < 0.001; ^‡^*p* < 0.0001.

At entry in the study, we found strong and significant effects of age and TIV (resp. GM volume) on WB, GM, and WM volumes (resp. HIP volume), both in men and in women (see Figure 4 and Tables 4–7). TIV and age showed the largest effect sizes of all covariates. On the contrary, age and TIV had much smaller effects on annualized rates of brain tissue losses: significant TIV effects were observed on the annualized rates of WB and GM in both sexes with a trend on HIP in men, whereas age effect was significant only on the annualized rates of WB and HIP loss in women with a trend on the GM volume in women (see Figure 5). Interestingly, when significant, the size of tobacco smoking effect was of the same order of magnitude as that of age (see Figures 1, 4).

Other covariates had fewer, usually less significant, and smaller effects than TIV and age.

WM lesion charge had similar effects in both sexes at entry in the study: positive on WM volume and negative on GM volume, resulting in no effect on WB volume. Regarding the annualized rates of tissues losses, WML charge was negatively correlated with the annualized rate of HIP volume loss.

Education negatively correlated with baseline WB and WM volumes in men as well as in women (see Tables 4, 6), but had no effect on annualized rates of tissue loss.

Male participants carrying ε 4 allele(s) had significantly higher baseline GM volumes than non-carriers, while having a reduced HIP baseline volume.

In men, alcohol consumption was associated with reduced baseline WB and WM volumes, while was positively associated with the annualized rate of WM loss. In women, alcohol increased baseline HIP volume and reduced annualized WB and GM loss.

Glycaemia had significant negative effects on baseline WB, GM, and WM volumes in men but not in women. However, it was associated with increased annualized WB and GM loss in women only.

The other covariates either had very few and small sized effects (BMI, CES-D) or no effect at all (MMSE, cholesterol, SBP and DBPs).

Pooling men and women resulted in very similar results regarding effects of covariates, the larger sample size helping some effects reaching the 0.05 significance level (see for example the negative effect of MMSE on baseline WM volume). In very few cases, where the covariate had opposite effects in men and women, discrepant results were observed between the pooled and the stratified analyses (see for instance the effect of ApoE-e4 on baseline GM volume, of alcohol on baseline HIP volume, or the effect of age on the longitudinal analysis of WB volume changes).

## Discussion

In a large sample of healthy elderly participants, we have found both sex-independent and sex-dependent effects of smoking on brain atrophy. In men, we observed reduced GM volumes at baseline and trends for increased annualized rate of WM and HIP loss. In women, we found reduced WM, and HIP volumes at baseline and increased annualized rate of HIP loss.

### Methodological issues

We have identified in the literature five cohort studies that used quantitative MRI for addressing the issue of tobacco-smoking impact on brain aging in healthy elderly individuals (Enzinger et al., 2005; Ikram et al., 2008; Debette et al., 2011; Durazzo et al., 2012; Hoogendam et al., 2012, see Table 10). Before comparing our findings with these previous reports, we think it is worthwhile listing the similarities and differences in the methodology implemented in these studies and in ours.

**Table 10:** **Main characteristics and findings of quantitative MRI past studies of tobacco-effects on brain atrophy**.

**Reference (cohort)**	**Design (N, delay)**	**Age**	**Smoking status**	**Brain phenotypes**	**Main findings**
Debette et al., 2011	Long	61	nS + fS: 1155 (85%)	GM + WM	cS: increased annual loss (resp. gain) in GM + WM (resp. THV)
(Framingham)	(1352, 6.3 years)		cS: 197 (15%)	THV	
Durazzo et al., 2012	Cross	76	nS: 118 (63%)	BREOS and AD ROIs	cS + fS: smaller SFG related to longer smoking duration
(ADNI)	(186)		fS: 63 (34%)		
			cS: 5 (3%)		
	Long		nS: 92 (64%)	BREOS and AD ROIs	cS + fS: greater atrophy rate smokers in BREOS and AD ROIs, but not in other GM ROI's or whole GM; greater loss in SFG related to longer smoking duration
	(144, 2 years)		fS + cS: 52 (46%)	Other GM ROIs, GM	
Enzinger et al., 2005	Cross	60	nS: 122 (61%)	GM + WM (in %TIV)	No effect of smoking
(ASPS)	(201)		fS + cS: 79 (39%)		
	Long				No effect of smoking
	(201, 6 years)				
Hoogendam et al., 2012	Cross	60	nS: 1154 (29%)	GM + WM	Reduced GM + WM volume in cS but not in fS
(Rotterdam)	(3962)		fS: 2076 (53%)		
			cS: 709 (18%)		
Ikram et al., 2008	Cross	73	nS: 211 (27%)	GM, WM, GM + WM	Reduced GM + WM volume in cS but not in fS
(Rotterdam)	(563)		fS: 265 (54%)		
			cS: 87 (18%)		

*Design: study design (cross: Cross-sectional, Long: longitudinal), sample size (N) and delay between the first and last MRI exams (for longitudinal design, in years). Age: mean participant's age at their first MRI exam. Smoking status: nS, fS, cS: never, former, current smokers. Brain phenotypes: gray matter (GM) and white matter (WM); THV, temporal horn volume; ROIs = regions of interest; BREOS, Brain Reward Executive Oversight System; AD, Alzheimer's disease; SFG, superior frontal gyrus*.

#### Cohort study design

The present study was based on a design allowing investigation of tobacco-related effects on atrophy both cross-sectionally in 1451 healthy older adults and longitudinally in 1111 of them after 4 years. Two previous studies (Enzinger et al., 2005; Durazzo et al., 2012) used the same kind of design, albeit on much smaller sample sizes (*N* = 186 including 144 at 2 year follow-up, and *N* = 201 at baseline and follow-up, respectively); two others were purely cross-sectional (Ikram et al., 2008; Hoogendam et al., 2012; *N* = 3952 and *N* = 490, respectively) and one reported only longitudinal data (Debette et al., 2011; *N* = 1352; 6 years follow-up). As regards the mean age of our sample, it falls within the range of previous studies sample mean age [60, 76].

#### Smoking consumption descriptors

All previous studies used categories similar to ours for describing the status of participants with respect to smoking, namely never, former or current smoker at the time of their participation in the study. In some studies, former smokers were pooled either with current smokers (Enzinger et al., 2005; Durazzo et al., 2012) or with non-smokers (Debette et al., 2011). With that respect, it is important to note that the number of current smokers in the (Durazzo et al., 2012) study was extremely small (5 to be compared to 58 former smokers) so findings of this study really concern former smokers which make them hardly comparable with those of Debette et al. (2011) established in a sample of 197 current smokers. In addition, previous studies lack details on individual smoking habits such as duration and quantity of smoking (pack-years), or dates when former smokers quit smoking. As a matter of fact, only one study (Durazzo et al., 2012) attempted at testing a dose dependent relationship between smoking and brain atrophy using lifetime smoking duration, a quantitative variable related to the number of pack-years used in our study.

#### Cerebral phenotypes

Here, in addition to WB volume, we studied the effect of tobacco separately for gray and WM, which has been done by only one of the previous studies (Ikram et al., 2008), whereas others preferred studying atrophy of either the WB volume (Enzinger et al., 2005; Debette et al., 2011; Hoogendam et al., 2012) or of sets of ad'hoc regions of interest (Durazzo et al., 2012). There are several reasons why GM and WM should be studied separately. First, their lifetime course are very different (Good et al., 2001; Abe et al., 2010), and some have suggested that their rate of atrophy during aging are very different (Smith et al., 2007). Second, the cellular content and vascular fraction of the two tissues are very different and one cannot thus a priori exclude the possibility of different pathophysiological effects of tobacco in each tissue. Third, GM and WM fraction are known to be different between men and women (Allen et al., 2003; Lemaître et al., 2005b; Leonard et al., 2008), which also calls for a separate analysis.

In addition to WB, GM, and WM we also investigated HIP atrophy, a target area for age-related disorders that was also analyzed by Durazzo et al. (2012) and by Debette et al. (2011), the latter using the temporal horn CSF volume as a surrogate marker for HIP volume.

#### Taking into account sex confounding effects

We believe sex segregation to be desirable for studies dealing with brain atrophy because of the potential confounding effect of sex due to differences between men and women in lifestyle, including tobacco consumption, and health parameters (Table 1). Such differences have indeed been shown by others to have significant sex-specific impact on brain aging in cognitively unimpaired elderly individuals (Franke et al., 2014). Thanks to the large sample size of the 3C-D-MRI cohort, we were able to assess tobacco-smoking effects separately in men and in women, while keeping high statistical power. Among previous studies, one made no mention at all of a sex effect (Enzinger et al., 2005), others included a sex effect when analyzing atrophy but did not investigate sex-dependent tobacco effects (Debette et al., 2011; Durazzo et al., 2012; Hoogendam et al., 2012), and one specifically mentioned testing interaction between sex and tobacco effects but did not report on it (Ikram et al., 2008). Actually, in the literature, we found only one study reporting tobacco impacts separately in men and in women, but the findings were based on a visual grading of brain atrophy (Longstreth et al., 2000).

In the present study, sex-segregated analysis allowed uncovering sex-specific of smoking on brain atrophy and notably that GM (resp. WM) was primarily affected by smoking in men (resp. in women). It also helped demonstrating that, despite similar trends, quantitative effects of smoking could be quite different in men and women, reaching significance in a subgroup but not in the other.

### Effect of tobacco consumption on whole brain volume

In our study, males who were still smoking after 65 had a strong trend of reduced baseline WB volume as compared to never smokers. This finding is in agreement with those of Ikram et al. (2008) and Hoogendam et al. (2012) who reported reduced WB volume in current smokers only. That such difference was not found in other cross-sectional studies (Enzinger et al., 2005; Durazzo et al., 2012) is likely to be due to the relatively small sample size and consequent lack of statistical power of these latter studies.

The present longitudinal analysis demonstrated that male current smokers had a very significant increase in their annualized rate of WB loss as compared to both never or former smokers, consistent with the findings of the Debette et al. (2011) study in which never and former smokers were pooled. Here, in addition, we also demonstrate that former smokers did not differ from never smokers in their WB rate atrophy, indicating that deleterious effects of smoking on brain atrophy stops when individuals quit smoking. Again, we believe that absence of similar findings in the Enzinger et al. (2005) longitudinal study may be attributed to a lack of statistical power or to the fact that they grouped former and current smokers in the same group.

In women, we did not find significant effects of smoking on WB baseline volume or annualized rate of loss. Here, a close look at the profiles of WB volumes at study entry across smoking status categories shows that effects of smoking on WB in women followed the same pattern as in men, albeit with smaller amplitude, but never reached significance. This is likely to be due to smaller smoking duration and intensity in women as compared to men, and also possibly to specific effects of hormone replacement therapy on brain atrophy.

Note that pooling men and women would have led us to conclude for a sex-independent effect of smoking on WB volume at study entry, similar what as reported by Ikram et al. (2008) and Hoogendam et al. (2012) studies, and also on its annualized rate of atrophy (Tables 8, 9). However, such pooling would have concealed sex-dependent specific effects of smoking not only on WB, but also on GM, WM, and HIP atrophy.

### Tissue- and sex-specific effects of smoking on gray and white matter volumes

As a matter of fact, as opposed to WB volume, effects of smoking on GM volume at entry time show distinct patterns between men and women. Baseline GM volume was modified by smoking in men, but not in women, the volume being lower in current smokers than in former or non-smokers. On the contrary, baseline WM volume was modified by smoking in women but not in men, the volume being again lower in current smokers than in former or never smokers. Among the five studies reported in Table 10, the study by Ikram et al. (2008) was the only one that investigated tissue-specific effects of smoking on baseline GM and WM volumes, reporting no significant smoking effect and no interaction between smoking and sex, only mentioning that “*current smoking was more related to total WM than GM*.” There have been other studies that investigated smoking effects on gray (Brody et al., 2004; Gallinat et al., 2006; Fritz et al., 2014) or WM (Yu et al., 2011; Fritz et al., 2014), but these studies reported effects on specific structures rather on whole tissue volumes. Thus, additional studies are needed in order to confirm and explain this sex-related difference in smoking effects on GM and WM. For both men and women, effects of smoking on tissue volumes were apparent in participants who were current smokers at the time of their entry of the study whereas former smokers seemed to be free of such tissue loss when compared to never smokers. Consequently grouping former and current smokers as done in Durazzo et al. (2012) and Enzinger et al. (2005) may have hidden those effects. This effect of smoking on WB volume at entry in current smokers only, also reported by others (Ikram et al., 2008; Hoogendam et al., 2012), is likely to be due to the longer duration and larger pack-years in the current smoker group, and suggests a dose threshold of tobacco consumption for its deleterious effects to take place.

Meanwhile, absence of smoking effect on the annualized GM atrophy rate in either men or women is in agreement with the only other similar report on this topic in which smoking status was found to affect specific GM ROI's but not GM as a whole (Durazzo et al., 2012). As for the annualized WM volume loss due to smoking, we observed a trend for a significant increase in men current smokers only, but a similar pattern was also observed in women. This suggests a possible effect in both sexes and future studies should include WM in analyses to assess this question.

These findings should also be interpreted within the framework of sexual brain dimorphism. In the present study, we found between sex differences regarding both baseline volumes and their rates of atrophy. Specifically, women exhibited larger WB annualized rate of atrophy than men, a finding that, to our knowledge, has never been reported before in such large sample of healthy elderly. We think that drastic changes in circulating hormone concentrations due to menopause in women could be one cause of such phenomenon. Indeed, a previous longitudinal study on a small sample of elderly women has shown that women under hormone replacement therapy (HRT) had smaller brain atrophy rates than those who were not under HRT (Raz et al., 2004). This finding was explained by the break in the neuroprotective effect of estrogens at menopause on the brain of women that did not take HRT (Gandy, 2003). In our study, more than 50% of the women longitudinal sample (406 among the 721) never had HRT which, we believe, explains in part the larger annualized rate of atrophy observed in the entire sample of women as compared to men.

### Effect of tobacco consumption on hippocampus volume

Baseline HIP volume was affected by smoking status in women only, female former smokers having a lower HIP volume than female never smokers. Moreover, a strong and very significant negative correlation was found between the number of pack-years and the HIP volume in female former smokers. Recall that in this analysis, GM volume was used as a covariate. Accordingly, observation of an absence of effect in men means that smoking has no specific effect on mean HIP as compared to its global effect on GM. In women, we did not find a smoking effect on GM as a whole: accordingly, observation of a smoking effect in women HIP volume means that this area is a site of specific vulnerability to tobacco smoking compared to global GM. To our knowledge, such a result has never been reported. Smoking effects on HIP volume have been rarely reported, the study of Durazzo et al. (2012) being the only one including the hippocampal area as an ROI of interest. However, in this study there was no significant effect on tobacco on baseline HIP, and a sex by smoking interaction was not searched for. In a more recent study in a sample of 82 young-to-middle-aged adults (Durazzo et al., 2013), the same authors did report lower total HIP volume (and subfields volumes) in smokers as compared to non-smokers but their sample included only 6 smoking women which did not allow testing for a sex effect. Other studies based on VBM (Brody et al., 2004; Gallinat et al., 2006; Yu et al., 2011) did not report effect of smoking on the HIP, but they were based on relatively small samples (*N* < 50) of younger participants (including young and middle aged adults), and did not search for a sex by smoking interaction.

As for the HIP annualized rate of atrophy, we observed a significant and strong effect in women with a similar trend in men, current smokers having larger rates of HIP loss, as compared to both former or never smokers. This result is consistent with the finding of Debette et al. (2011) of accelerated temporal horn volume increase in current smokers as opposed to never and former smokers, as well as with the report of Durazzo et al. (2013) showing larger rates of HIP loss in current smokers as compared to non-smokers. It is however in partial disagreement with other findings (Durazzo et al., 2012) reporting accelerated volume loss of medial temporal GM structures in a group of 52 mainly former smokers, whereas our study indicated no difference in HIP annualized rate of atrophy between non and former smokers. We think that methodological factors are likely to explain this discrepancy.

Overall, our results emphasize HIP as particularly vulnerable to tobacco smoking, as it is to many adverse conditions such as cerebral ischemia, epileptic seizures, and oxidative stress, Wang and Michaelis (2010). Underlying mechanisms for such vulnerability are not well-determined (particularly their causality relationships), but reactive oxygen species known to be abundant in the mainstream of cigarette smoke (Pryor et al., 1993), appear to be potential candidates.

### Other covariates

As this paper focuses on tobacco smoking and atrophy, we will briefly discuss the largest and most significant effects of these covariates on brain atrophy. Besides, we previously reported elsewhere on effects on brain atrophy of ApoE ε 4 genotype (Lemaître et al., 2005b; Crivello et al., 2010), depression (Elbejjani et al., 2014), and WM lesion (Godin et al., 2009). Note also that, with the exception of age and TIV, the size of effects of covariates were usually smaller that those of smoking.

#### Alcohol

In our study, alcohol was associated with increased WB atrophy, being significant on the WB and WM of men only. These findings fit with previous reports showing alcohol detrimental effect on brain tissue atrophy in both sexes (Hommer et al., 2001; Mukamal et al., 2001; Demirakca et al., 2011), or more marked in men than in women (Pfefferbaum et al., 2001; Anstey et al., 2006). As regards to the HIP, the trend for increased atrophy associated to alcohol agrees with other studies (Harding et al., 1997; Pfefferbaum et al., 2001; Anstey et al., 2006; Demirakca et al., 2011). Note that in our study the effect was observed in men only, which can be explained by the larger alcohol intake in men. Actually, women were found to have reduced HIP atrophy as compared to global GM, which may be mediated by the reduction of cardiovascular risk factors in low level of alcohol intake individuals (Tolstrup et al., 2006). When pooling men and women, our longitudinal analysis showed no significant effect of alcohol intake on the annualized rate of brain atrophy, in apparent contradiction with previous findings of accelerated brain atrophy in heavy drinkers (Akiyama et al., 1997; Enzinger et al., 2005). We think that this discrepant result is explained by the relatively low alcohol intake in our cohort (1.5 and 0.5 drink per day in men and women, respectively).

#### Glycaemia

In our study, increased glycaemia was associated with increased atrophy of both gray and WM, the association being significant in men only. These results are consistent with previous reports enhanced atrophy of WB with increased glycaemia (Araki et al., 1994; Ikram et al., 2008; Hoogendam et al., 2012) or percentage of glycated hemoglobin A (HbA_1c_) (Enzinger et al., 2005). Longitudinally, we found higher glycaemia associated with a larger rate of GM atrophy that was significant in women only, a finding in line with that of another longitudinal study (Enzinger et al., 2005) reporting association a higher rate of brain atrophy with HbA_1c_.

#### Body mass index

BMI was positively associated with WM volume, but not with GM, the association being significant in women only. These findings are in agreement with a previous report of increased WM volume in obese adults, this effect being partially reversed by dieting (Haltia et al., 2007). Absence of BMI effect on GM atrophy has also been reported others (Haltia et al., 2007; Debette et al., 2014). Note, however, that two recent studies reported discrepant results, namely increase of the Brain Age score (an index of accelerated cortical aging) with BMI especially in men (Franke et al., 2014), reduced GM atrophy with increased BMI (Bobb et al., 2012). But those differences might be explained by the covariates used in both studies, especially with Bobb et al. (2012) which did not include a sex effect.

As regards the longitudinal analyses, we did not find any change in annualized rates of GM, WM or HIP loss, neither in men nor in women. This is in agreement with the findings reported by Debette et al. (2011), but others have reported partially conflicting findings, namely increased rate of WB atrophy with increased BMI (Enzinger et al., 2005) or reduced rate of WM atrophy with increased BMI (Bobb et al., 2012).

#### Education

Education was negatively associated with WM, but not with GM, in both sexes, in agreement with previous reports of higher education individuals having increased sulcal CSF volume (Coffey et al., 1999; Longstreth et al., 2000) and thinner cortex in temporal, occipital and parietal areas as well as lower HIP volume (Pillai et al., 2012). Education had, however, not effect on the annualized rates of brain tissue loss. Our result thus argues in favor of the hypothesis stating that education might influence cognitive reserve through connectivity and/or synapses efficiency rather than by neuron numbers.

### Conflict of interest statement

The authors declare that the research was conducted in the absence of any commercial or financial relationships that could be construed as a potential conflict of interest.
